# Case Report: Fatal varicella-zoster virus meningoencephalitis with Froin-like cerebrospinal fluid and hydrocephalus following pembrolizumab-containing chemoimmunotherapy

**DOI:** 10.3389/fphar.2026.1937378

**Published:** 2026-10-07

**Authors:** Fangfang Shen, Siyuan Fan, Yanyan Wang, Yan Xu, Wei Guo

**Affiliations:** 1 Department of Respiratory Medicine, Shanxi Province Cancer Hospital/Shanxi Hospital Affiliated to Cancer Hospital, Chinese Academy of Medical Sciences/Cancer Hospital Affiliated to Shanxi Medical University, Taiyuan, China; 2 Department of Neurology, Peking Union Medical College Hospital, Chinese Academy of Medical Sciences and Peking Union Medical College, Beijing, China; 3 Department of Radiology, Shanxi Province Cancer Hospital/Shanxi Hospital Affiliated to Cancer Hospital, Chinese Academy of Medical Sciences/Cancer Hospital Affiliated to Shanxi Medical University, Taiyuan, China; 4 Department of Respiratory and Critical Care Medicine, Peking Union Medical College Hospital, Chinese Academy of Medical Sciences, Peking Union Medical College, Beijing, China

**Keywords:** adverse drug reaction, dysregulated immunity, immune checkpoint inhibitors, neurotoxicity, pharmacovigilance, varicella-zoster virus

## Abstract

**Background:**

In patients receiving immune checkpoint inhibitors (ICIs), central nervous system (CNS) infections may mimic or coexist with neurological immune-related adverse events (irAEs). ICI-driven immune dysregulation has been proposed to amplify inflammation against latent pathogens (“immunotherapy infections due to dysregulated immunity”, ITI-DI), but this remains a hypothesis-generating framework rather than a validated diagnosis.

**Case Presentation:**

A 54-year-old man with stage IIIC lung adenocarcinoma developed a herpes zoster eruption and lumbosacral pain 7 days after the first infusion of pembrolizumab-containing chemoimmunotherapy, followed by rapidly progressive flaccid paraplegia, urinary incontinence, and declining consciousness. Intravenous acyclovir was not initiated until 20 days after rash onset. The first cerebrospinal fluid (CSF) sample was xanthochromic and coagulated spontaneously in the collection tube (Froin-like CSF), precluding protein measurement. A second lumbar puncture showed mild pleocytosis (8 cells/ μ L; 70% neutrophils) and undetectable glucose; varicella-zoster virus (VZV) DNA was detected in CSF by post-mortem targeted polymerase chain reaction. Spinal Magnetic resonance imaging (MRI) showed diffuse meningeal enhancement; follow-up cranial imaging showed acute hydrocephalus. Despite intravenous acyclovir, corticosteroids, and intravenous immunoglobulin, the patient died 43 days after pembrolizumab initiation.

**Conclusion:**

This case highlights VZV CNS infection as an important diagnostic consideration in ICI-treated patients who develop herpes zoster and neurological symptoms, because infection may mimic or coexist with a neurological irAE. The temporal relationship with pembrolizumab raises, but does not establish, the possibility that PD-1 blockade modified the inflammatory response. Prompt virological testing and adequately dosed antiviral therapy are central to management; the role of adjunctive immunomodulation remains uncertain and should be individualized.

## 1 Introduction

Monoclonal antibodies targeting the programmed cell death protein 1 (PD-1)/programmed cell death ligand 1 (PD-L1) axis are standard of care for various advanced malignancies, including non-small cell lung cancer (NSCLC) (Ribas and Wolchok, 2018; Reck, 2018). However, the same pharmacological abrogation of immune tolerance that confers anti-tumor efficacy also induces immune-related adverse events (irAEs) across diverse organ systems (Ramos-Casals et al., 2020; Keam et al., 2024) and may disrupt host infectious homeostasis: it has been proposed that the disinhibited immune system can amplify pathological inflammation against latent or opportunistic pathogens—a concept termed “immunotherapy infections due to dysregulated immunity” (ITI-DI) (Morelli et al., 2022; Schönberger et al., 2025). This framework remains hypothesis-generating rather than a validated diagnostic entity.

Among ICI-induced toxicities, neurological irAEs (neuro-irAEs) are comparatively rare but carry disproportionately high morbidity and mortality (Postow et al., 2018; Cuzzubbo et al., 2017) and can manifest unpredictably along the entire neuraxis (Fan et al., 2020). A major pharmacovigilance challenge is the extensive phenotypic overlap between true neuro-irAEs and primary central nervous system (CNS) infections, which forces clinicians to balance prompt, adequately dosed antiviral therapy against the uncertain role of adjunctive immunomodulation.

Here, we report a patient with advanced lung adenocarcinoma who developed fatal varicella-zoster virus (VZV) meningoencephalitis with Froin-like cerebrospinal fluid and hydrocephalus shortly after starting pembrolizumab-containing chemoimmunotherapy. We discuss the differential diagnosis between CNS infection and neurological irAE and present, explicitly as a hypothesis, the possibility that PD-1 blockade modified the inflammatory response to VZV reactivation.

## 2 Case description

A 54-year-old man presented with cough, right shoulder and back pain, facial edema, and chest-wall varicosities, and was diagnosed with poorly differentiated stage IIIC (T4N3M0, eighth edition) lung adenocarcinoma with cervical lymph node metastasis, right pleural effusion, and superior vena cava syndrome; molecular testing showed TP53 mutation, high tumor mutational burden, and a PD-L1 tumor proportion score of 10%. Throughout this report, day 0 denotes the day of the first (and only) pembrolizumab infusion. He received two cycles of pemetrexed (500 mg/ ${\text{m}}^{2}$ ) plus carboplatin (AUC = 5), followed by progressive disease. Second-line nanoparticle albumin-bound (nab)-paclitaxel (260 mg/ ${\text{m}}^{2}$ ) was then given, and on day 0 he received nab-paclitaxel (260 mg/ ${\text{m}}^{2}$ ) with the first—and only—dose of pembrolizumab 200 mg.

Baseline investigations before chemoimmunotherapy showed a white-cell count of $5.06\times 10^{9}$ /L (absolute neutrophils $3.38\times 10^{9}$ /L, absolute lymphocytes $0.97\times 10^{9}$ /L), platelets $286\times 10^{9}$ /L, alanine aminotransferase 17 U/L, aspartate aminotransferase 15 U/L, serum albumin 40 g/L, and creatinine 61 μ mol/L. HIV testing was negative; he had no relevant comorbidities and no prior or concomitant systemic corticosteroid or immunosuppressive exposure (no corticosteroid premedication was given with nab-paclitaxel). He reported no history of varicella, herpes zoster, or VZV vaccination; baseline VZV serology was not performed.

On day 7, a pruritic erythematous rash involving the left hemithorax and axilla appeared, which initially received no medical attention and no antiviral treatment; at the same time the patient developed lumbosacral pain that progressively worsened despite oral diclofenac–codeine, disturbing sleep and walking. On day 27 he was admitted; the predominant complaint was lumbosacral pain. He was ambulatory, fully conscious, and without meningism. By then the eruption involved multiple dermatomes (T2–T4), with scattered erythema, erosions and crusting accompanied by pain and pruritus, and was diagnosed as herpes zoster; intravenous acyclovir (10 mg/kg every 8 h) was started. Magnetic resonance imaging (MRI) showed diffuse thickening of the cervical, thoracic, and lumbar meninges, and chest computed tomography (CT) showed a partial response according to RECIST. The complete clinical course and interventions are summarized in Figure 1 and Table 1.

**FIGURE 1:**
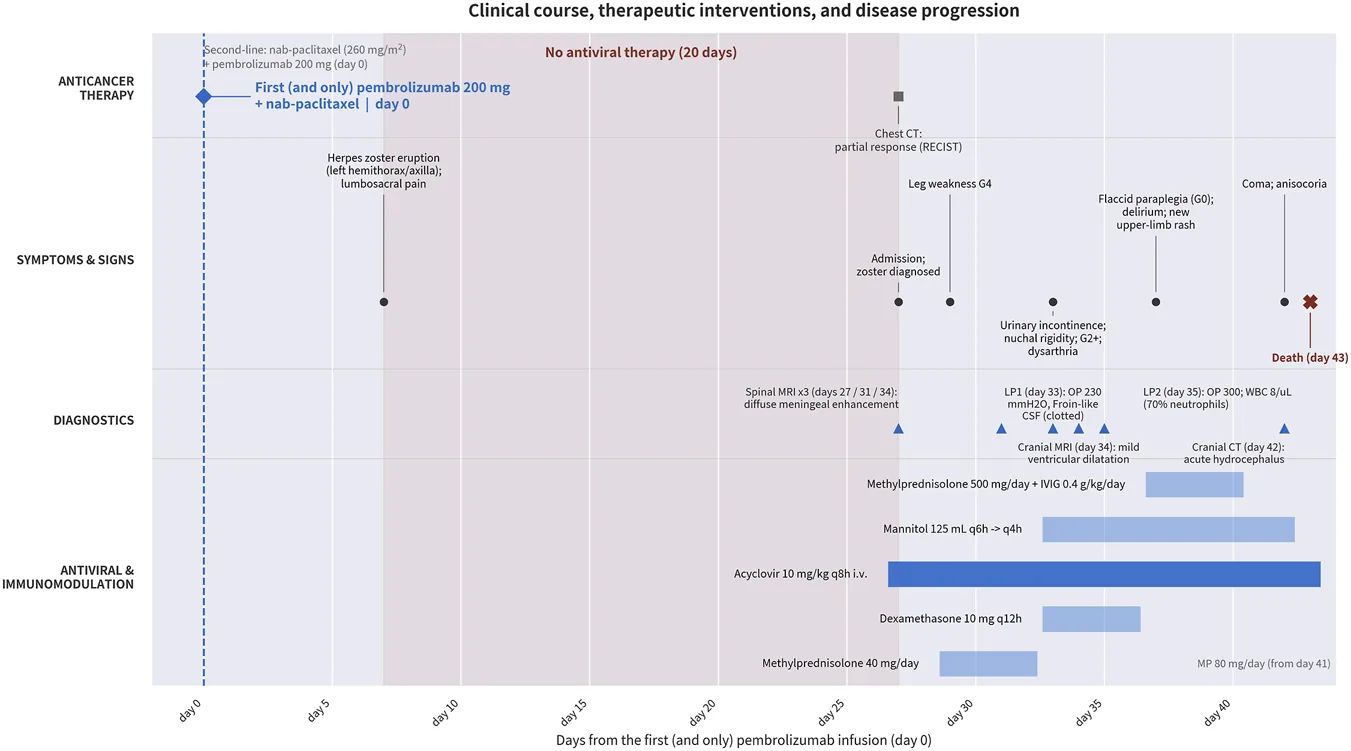
Timeline of the patient’s clinical course, therapeutic interventions, and disease progression. Day 0 denotes the first (and only) pembrolizumab infusion. The chronological sequence details the pembrolizumab infusion (day 0), the herpes zoster eruption and lumbosacral pain (day 7), admission and initiation of antiviral therapy (day 27), the progressive neurological decline, the 20-day interval without antiviral therapy after rash onset, antiviral and immunomodulatory treatment (with doses), both lumbar punctures, the development of hydrocephalus, and the fatal outcome (day 43). Abbreviations: PR, partial response; RECIST, Response Evaluation Criteria in Solid Tumours; nab-paclitaxel, nanoparticle albumin-bound paclitaxel; MP, methylprednisolone; DXM, dexamethasone; IVIG, intravenous immunoglobulin; LP, lumbar puncture; MRI, magnetic resonance imaging; CT, computed tomography; OP, opening pressure; CSF, cerebrospinal fluid; VZV, varicella-zoster virus; PCR, polymerase chain reaction.

**TABLE 1:** Serial laboratory, neurological, and treatment data.

Parameter	Baseline	Day 29	Day 33	Day 35	Day 42
Peripheral blood					
WBC ( $\times 10^{9}$ /L)	5.06	4.05	4.83	6.56	4.3
ANC ( $\times 10^{9}$ /L)	3.38	2.97	3.72	5.54	4.2
ALC ( $\times 10^{9}$ /L)	0.97	0.72	0.52	0.52	0.10
Platelets ( $\times 10^{9}$ /L)	286	286	291	293	128
Hemoglobin (g/L)	NA	115	122	130	113
Na/K/Cl (mmol/L)	NA	130/3.46/86	132/3.71/93	128/4.90/86	132/3.35/97
ALT/AST (U/L), alb (g/L), Cr ( μ mol/L)	17/15, 40, 61	NA	NA	25/24, 39.4, 55	36/29, 28.2, 32
Neurological examination					
Consciousness	Alert	Alert	Alert	Intermittently impaired	Delirium → coma (day 42)
Lower-limb strength (MRC)	5/5	4/5	2+/5	0/5 (day 37)	0/5
Other findings	—	—	Urinary incontinence, nuchal rigidity, dysarthria (day 33)	—	New upper-limb rash (day 37); anisocoria (day 42)
Treatment					
Acyclovir	—	10 mg/kg q8 h i.v. (started day 27)	Continued	Continued	Continued until death (day 43)
Corticosteroid	—	MP 40 mg/day (days 29–32)	DXM 10 mg q12 h (days 33–36)	DXM continued	MP 500 mg/day (days 37–40); 80 mg/day (from day 41)
IVIG	—	—	—	—	0.4 g/kg/day (days 37–40)
Mannitol	—	—	125 mL q6 h (started day 33)	Continued	125 mL q4 h

NA, not available; ANC, absolute neutrophil count; ALC, absolute lymphocyte count; MP, methylprednisolone; DXM, dexamethasone; IVIG, intravenous immunoglobulin.

On day 29, symmetric lower-limb weakness appeared (MRC grade 4/5); because a neurological immune-related adverse event could not be excluded, intravenous methylprednisolone 40 mg/day was given on days 29–32. On day 33, urinary incontinence developed, lower-limb strength declined to grade 3/5, nuchal rigidity appeared, strength further declined to grade 2+/5, and new dysarthria developed. An emergent lumbar puncture (LP) the same day yielded an opening pressure of 230 ${\text{mmH}}_{2}$ O and pale-yellow, xanthochromic cerebrospinal fluid (CSF) that was gelatinous and coagulated spontaneously within the collection tube (Figure 2). Because the sample coagulated, routine biochemical analysis and cell counting could not be performed; cytological examination showed a few lymphocytes and no malignant cells. Because no quantitative protein measurement and no radiological evidence of a focal spinal subarachnoid block were obtained, we refer to these findings—compatible with Froin’s syndrome—as Froin-like CSF throughout. Nerve conduction studies and electromyography were not performed. Muscle tone, deep tendon reflexes, plantar responses, coordination, and a formal sensory level were not consistently documented in the available records.

**FIGURE 2:**
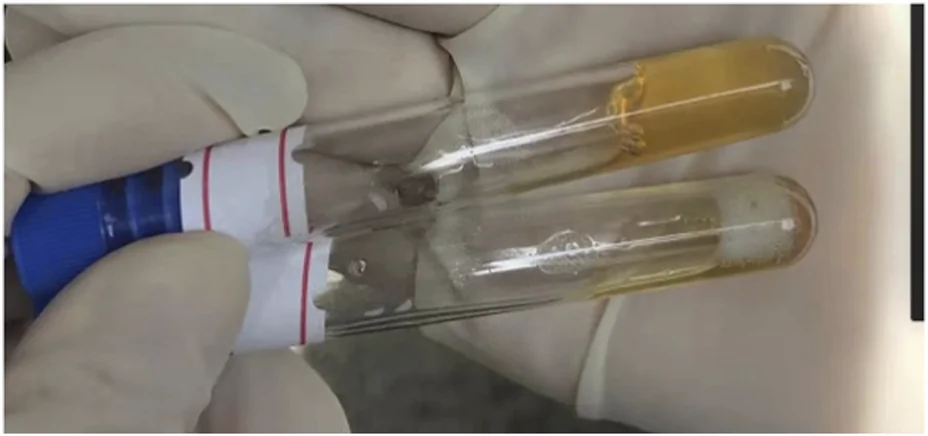
Macroscopic appearance of the first cerebrospinal fluid sample (day 33). The xanthochromic CSF was gelatinous and coagulated spontaneously within the collection tube (Froin-like CSF), precluding quantitative protein measurement, routine biochemical analysis, and cell counting.

## 3 Diagnostic assessment, therapeutic interventions, follow-up and outcomes

Given the abnormal CSF appearance and an opening pressure of 230 ${\text{mmH}}_{2}$ O, on days 33–36 corticosteroids were changed to intravenous dexamethasone 10 mg every 12 h, and intravenous mannitol 125 mL every 6 h was started for suspected raised intracranial pressure; renal function remained normal (creatinine 65 μ mol/L) and no dose adjustment was required. Intravenous fluids and electrolyte correction were given (serial electrolytes in Table 1). Skin-lesion VZV polymerase chain reaction (PCR) was not performed; antiviral resistance or an alternative agent was not considered given the rapid progression.

A repeat LP on day 35 yielded an opening pressure of 300 ${\text{mmH}}_{2}$ O and pale-yellow, clear CSF without clot formation. Cell counting showed mild pleocytosis ( $8\times 10^{6}$ /L, i.e., 8 cells/ μ L; reference 0–5) with 70% neutrophils, 20% lymphocytes, and 10% monocytes, and a red-cell count of $2,060\times 10^{6}$ /L; without sequential tube counts or spectrophotometry, a traumatic tap cannot be formally excluded, although the xanthochromia is also compatible with prior hemorrhage or markedly elevated CSF protein. CSF glucose was repeatedly below the lower limit of detection (hexokinase method); CSF chloride was 91 mmol/L (reference 120–132 mmol/L). The Pandy test was strongly positive (+++), indicating marked hyperproteinorrhachia.

India-ink/alcian-blue staining showed no cryptococcus and no acid-fast bacilli. Cytology of this second sample showed occasional large nucleated cells but no definite malignant cells, as had cytology of the first. CSF metagenomic next-generation sequencing (mNGS) detected no viral, bacterial, mycoplasmal, chlamydial, fungal, or parasitic sequences. CSF anti-VZV IgG, intrathecal antibody synthesis testing, oligoclonal bands, and IgG index were not performed. A 14-item paraneoplastic antibody panel (anti-Hu [HuD/HuC/HuB], Yo, Ri [Nova1/Nova2], CV2, PNMA2/Ma2, amphiphysin, Tr, Zic4, GAD65, recoverin, titin, SOX1, Ma1, and PKC γ IgG; immunoblot) was negative, as were anti-NMDA receptor IgG (cell-based assay, CSF and serum) and anti-AQP4, anti-MOG, and anti-GFAP IgG (cell-based assay).

Serial spinal MRI during this period (lumbar spine, day 27; thoracic spine, day 31; cervical spine, day 34; all 3.0-T, plain and contrast-enhanced) showed diffuse linear meningeal enhancement along the cervical, thoracic, and lumbar thecal sac (Figure 3A), without spinal metastatic disease, cord compression, or radiological evidence of a spinal CSF block; the radiology reports did not describe cord T2 hyperintensity, cord swelling, or nerve-root/cauda equina enhancement.

**FIGURE 3:**
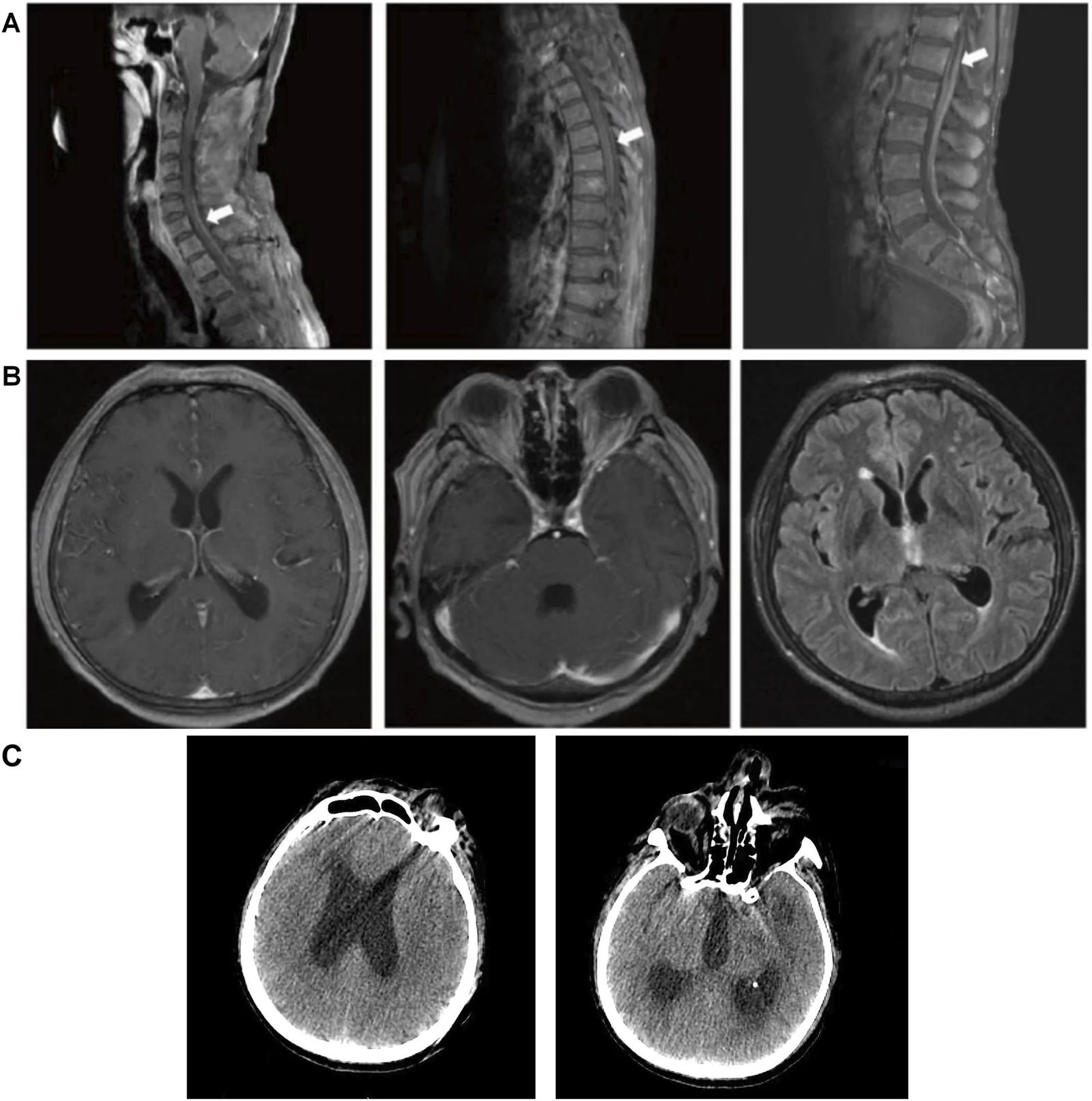
Imaging of the spine and brain. **(A)** Sagittal post-contrast spinal magnetic resonance imaging (MRI; 3.0-T, plain and contrast-enhanced; lumbar spine day 27, thoracic spine day 31, cervical spine day 34) demonstrating diffuse linear meningeal enhancement along the cervical, thoracic, and lumbar thecal sac; arrows indicate the thickened, enhancing meninges. **(B)** Cranial MRI (3.0-T, plain and contrast-enhanced; day 34; axial post-contrast T1-weighted and FLAIR images) demonstrating mild dilatation of the lateral, third, and fourth ventricles, without infarction, hemorrhage, encephalitic lesion, or cranial meningeal enhancement. **(C)** Plain cranial computed tomography (CT) on day 42 (two axial slices) demonstrating marked interval progression with prominent dilatation of the lateral and third ventricles, consistent with acute hydrocephalus. The spinal images show no metastasis, cord compression, or radiological evidence of a focal CSF block.

Cranial MRI on day 34 (3.0-T, plain and contrast-enhanced) showed, on direct comparison with the baseline study, mild dilatation of the lateral, third, and fourth ventricles (Figure 3B); no infarction, hemorrhage, or encephalitic lesion was identified; there was no cranial meningeal enhancement; and transependymal CSF flow was not commented on.

These findings supported a clinical and radiological diagnosis of VZV meningoencephalitis with suspected myeloradicular involvement and secondary hydrocephalus; without biopsy, autopsy, or vascular imaging (CTA/MRA, catheter angiography, or vessel-wall imaging), VZV vasculopathy and tissue necrosis could not be established. Serial laboratory, neurological, and treatment data are in Table 1, and CSF findings are in Table 2.

**TABLE 2:** Cerebrospinal fluid (CSF) findings from the two lumbar punctures.

Parameter	Day 33 (LP1)	Day 35 (LP2)
OP ( ${\text{mmH}}_{2}$ O)	230	300
Appearance	Pale yellow, gelatinous, clotted	Pale yellow, clear, no clot
Total protein	NP (clotted)	Pandy +++ (qualitative)
Glucose	NP	Below detection limit
Chloride (mmol/L)	NP	91 (ref. 120–132)
Leukocytes ( $\times 10^{6}$ /L)	NP	8 (70% N, 20% L, 10% M)
Red cells ( $\times 10^{6}$ /L)	NP	2,060
Microbiology	NP	Cryptococcus neg.; AFB neg.; mNGS all neg.; VZV PCR pos. (post-mortem, qualitative)
Cytology	Few lymphocytes; no malignant cells	Occasional large nucleated cells; no definite malignant cells

LP, lumbar puncture; OP, opening pressure; NP, not performed; mNGS, metagenomic next-generation sequencing; VZV, varicella-zoster virus; PCR, polymerase chain reaction.

On day 37, lower-limb strength reached grade 0/5 (flaccid paraplegia), with marked delirium and agitation and a new patchy erythematous rash on both upper limbs. Given the refractory course and suspicion of a severe immune-mediated component, pulsed intravenous methylprednisolone 500 mg/day and intravenous immunoglobulin (IVIG; 0.4 g/kg per day) were given on days 37–40, with mannitol increased to 125 mL every 4 h; acyclovir was continued until death. During treatment he transiently improved (rousable, with irrelevant verbal responses, intermittent drinking, and clearer articulation). Because multiple treatments were administered simultaneously, this improvement is reported descriptively and cannot be attributed to any single intervention or mechanism.

On day 41, methylprednisolone was reduced to 80 mg/day. On day 42, the patient progressed to coma with anisocoria (left pupil 4 mm, right pupil 3 mm) and absent pain and light reflexes; plain cranial CT showed marked dilatation of the lateral, third, and fourth ventricles, indicating hydrocephalus (Figure 3C). No neurosurgical consultation was obtained, intracranial pressure was not invasively monitored, and no CSF diversion (external ventricular drainage, lumbar drainage, or shunt), mechanical ventilation, or other intensive-care intervention was performed. The family declined invasive resuscitation. The patient died on day 43. Post-mortem targeted PCR for VZV DNA in CSF (Neurology Laboratory, Peking Union Medical College Hospital) was positive. The immediate cause of death was not formally established; the course is consistent with cerebral herniation complicating acute hydrocephalus, but the contributions of hydrocephalus, ongoing CNS infection, and withdrawal of invasive support cannot be separated retrospectively.

## 4 Discussion

This case illustrates the diagnostic difficulty when herpes zoster and progressive neurological symptoms follow immune checkpoint inhibitor (ICI) therapy: the picture may reflect severe varicella-zoster virus (VZV) central nervous system (CNS) infection, a neurological immune-related adverse event (irAE), or both. ICIs have been proposed to potentiate tissue injury through unregulated, antigen-driven immune hyperactivation rather than generalized immunosuppression (Liu et al., 2020), and atypical reactivation of latent pathogens (*Mycobacterium tuberculosis* (Shi et al., 2021), VZV (Shionoya et al., 2025)) and opportunistic bacterial infections (Lasagna et al., 2022) have been reported during ICI therapy. Here, the temporal proximity to the first pembrolizumab-containing cycle raises the possibility that PD-1 blockade modified the inflammatory response to VZV reactivation; however, equally plausible contributors cannot be distinguished in a single case: concomitant cytotoxic chemotherapy, active malignancy, baseline lymphopenia (absolute lymphocyte count $0.97\times 10^{9}$ /L before chemoimmunotherapy, declining to $0.52\times 10^{9}$ /L during the neurological illness and to $0.10\times 10^{9}$ /L on day 42—although this terminal decline followed high-dose corticosteroid therapy and cannot be attributed to the disease itself), and a 20-day interval between rash onset and intravenous acyclovir initiation.

Leptomeningeal metastasis is an important alternative or coexisting diagnosis given the lung adenocarcinoma, diffuse spinal meningeal enhancement, low CSF glucose, and hydrocephalus. Arguing against it, CSF cytology from both lumbar punctures showed no malignant cells, imaging showed no nodular leptomeningeal disease, and the tumor burden was simultaneously responding to chemoimmunotherapy (partial response on chest CT, day 42); nevertheless, cytology used single samples of unstated volume without repeat cytology or flow cytometry, so leptomeningeal metastasis cannot be definitively excluded. Tuberculous and fungal meningitis were not supported: no acid-fast bacilli or cryptococcus were seen, and CSF metagenomic next-generation sequencing (mNGS) detected no bacterial, mycobacterial, or fungal sequences. Notably, mNGS failed to detect VZV whereas targeted PCR (performed post-mortem) was positive, consistent with the lower sensitivity of mNGS for VZV; a negative mNGS result does not exclude VZV CNS infection. Other alternatives include bacterial meningitis (unlikely given the mild pleocytosis and negative microbiology, although Gram stain and culture were unavailable), infectious arachnoiditis, ICI-associated aseptic meningitis or myelitis, compressive spinal disease (excluded on MRI), and VZV myeloradiculitis without vasculopathy. CSF VZV DNA supports VZV CNS involvement but does not exclude a concurrent immune-mediated process, and the syndrome cannot be localized with certainty: flaccid paraplegia with sphincter dysfunction may reflect myelitis, myeloradiculitis, cauda equina involvement, or polyradiculopathy (nerve conduction studies were not performed).

The Froin-like CSF and strongly positive Pandy test are compatible with marked hyperproteinorrhachia, but protein could not be quantified and no spinal subarachnoid block was demonstrated; Froin’s syndrome also occurs with infection, neoplasia, inflammatory arachnoiditis, and mechanical obstruction, so it cannot prove a pembrolizumab-driven process or blood–spinal cord barrier failure. Similarly, the elevated red-cell count with xanthochromia may reflect prior hemorrhage or markedly elevated CSF protein and does not by itself establish VZV vasculopathy; without vascular imaging or CSF anti-VZV IgG, we regard vasculopathy as a possible mechanism rather than an established diagnosis (Nagel et al., 2007; Gilden et al., 2009). The isolated low CSF chloride is nonspecific and is therefore reported only as part of the complete CSF dataset without further interpretation; the mild neutrophil-predominant pleocytosis is atypical for uncomplicated viral meningitis but likewise nonspecific.

ICI-treated patients have been proposed to develop infections in which dysregulated, pathogen-directed immunity amplifies tissue injury (the ITI-DI framework) (Morelli et al., 2022; Shionoya et al., 2025). In our patient, no CSF cytokine analysis, immune-cell phenotyping, VZV-specific T-cell study, clonality analysis, biopsy, or autopsy was performed; immune amplification therefore cannot be established. We present the analogy with immune reconstitution inflammatory syndrome (IRIS) (Müller et al., 2010)—pharmacological disinhibition of pathogen-specific cellular immunity precipitating aberrant inflammation against latently infected tissues—strictly as a possible explanatory model, not as demonstrated pathogenesis. PD-L1 blockade potentiates the effector capacity of VZV-specific CD $8^{+}$ T-cells experimentally (Jones et al., 2019), a hyperinflammatory environment has been reported in ICI-associated VZV reactivation (Shionoya et al., 2025), and fatal ICI-associated encephalitis with prominent local inflammation has been described (Johnson et al., 2019; Lopes et al., 2025). The transient improvement after pulse methylprednisolone and IVIG is compatible with, but does not prove, an immune-mediated component, as it followed multiple simultaneous treatments and may represent natural fluctuation. The negative antibody panels (paraneoplastic, anti-NMDAR, anti-AQP4/MOG/GFAP) narrow the differential but neither exclude a neurological irAE nor demonstrate a T-cell-mediated mechanism.

Evidence against this hypothesis should also be acknowledged: in a nationwide Korean cohort, herpes zoster incidence was lower among ICI-treated than non-ICI-treated patients (Jung et al., 2024). Although this does not exclude rare severe immune-amplified infections, a single case cannot establish a general causal relationship between PD-1 blockade and VZV reactivation.

This is not the first demonstration that VZV can cause Froin’s syndrome, necrotizing vasculitis, or encephalomyelitis: a pathologically documented fatal case with all three features was reported before the ICI era (Kleinschmidt-DeMasters et al., 1998), so this combination is not specific to immune checkpoint inhibition. The value of the present report lies in the unusually severe VZV CNS syndrome occurring in close temporal proximity to pembrolizumab and the resulting diagnostic difficulty between infection and neurological immune toxicity.

With respect to management, the most actionable lesson of this case is the cost of delayed antiviral therapy. The zoster eruption appeared on day 7 but received no medical attention; herpes zoster was diagnosed only at readmission on day 27, when intravenous acyclovir was initiated—20 days after rash onset—by which time myelopathic signs were already emerging. Whether earlier antiviral treatment would have changed the outcome is unknowable, but this delay is a plausible, preventable contributor to progression and reinforces the principle of prompt, adequately dosed antiviral therapy whenever herpes zoster is recognized in an ICI-treated patient. The administered dose (10 mg/kg every 8 h) fell within the 10–15 mg/kg every 8 h recommended for central nervous system VZV infection (Tunkel et al., 2008); the correctable factor in this case was therefore the delay in recognition and treatment initiation rather than antiviral dosing. Conversely, this case cannot support early concurrent high-dose corticosteroids: corticosteroids were escalated and IVIG administered, yet the patient died, and potent immunosuppression during active VZV infection carries important risks (Esfahani et al., 2020; Blum et al., 2023). The role of adjunctive immunomodulation remains uncertain; if an immune-mediated component is strongly suspected, it may be considered individually, with adequate antiviral therapy and multidisciplinary assessment. Further research is needed to identify which patients, if any, might benefit from early adjunctive immunomodulation.

### 4.1 Limitations

This is a retrospective report of a single fatal case, and several investigations were not performed: quantitative CSF protein measurement (precluded by spontaneous coagulation of the first sample), CSF albumin and lactate, a simultaneous serum glucose sample, sequential tube red-cell counts and spectrophotometry, Gram stain and bacterial culture, vascular imaging, detailed spinal cord signal assessment (cord T2 signal, cord swelling, nerve-root enhancement, and spinal diffusion imaging were not described or documented), CSF anti-VZV IgG and intrathecal antibody synthesis, quantitative VZV PCR, skin-lesion PCR, CSF cytokine analysis, immune-cell phenotyping, VZV-specific T-cell and clonality studies, nerve conduction studies and electromyography, repeat CSF cytology with larger volumes or flow cytometry, and autopsy. These absences preclude definitive mechanistic characterization and leave residual uncertainty, particularly regarding leptomeningeal metastasis and VZV vasculopathy; where information was unavailable, we have stated this explicitly rather than substituting inference.

## 5 Conclusion

We report a fatal case of VZV meningoencephalitis with Froin-like cerebrospinal fluid and hydrocephalus following pembrolizumab-containing chemoimmunotherapy in a patient with advanced NSCLC. This case highlights VZV central nervous system infection as an important diagnostic consideration in patients receiving immune checkpoint inhibitors who develop herpes zoster and neurological symptoms, because infection may mimic or coexist with a neurological immune-related adverse event. The temporal relationship with pembrolizumab raises, but does not establish, the possibility that PD-1 blockade modified the inflammatory response. Prompt virological testing and adequately dosed antiviral therapy are central to management, and delays in antiviral initiation are a plausible, preventable contributor to progression. The role of adjunctive immunomodulation remains uncertain and should be individualized through multidisciplinary neurological and infectious-disease assessment.

## 6 Patient perspective

The patient died during the same hospitalization, precluding post-discharge follow-up or a direct patient perspective, and a family perspective could not be obtained. We report this case to assist the early recognition and management of similar life-threatening events.

## Data Availability

The original contributions presented in the study are included in the article/Supplementary Material, further inquiries can be directed to the corresponding authors.
